# *ERBB2/ ERBB3*-mutated S100/ SOX10-positive unclassified high-grade uterine sarcoma: first detailed description of a novel entity

**DOI:** 10.1007/s00428-024-03908-3

**Published:** 2024-08-28

**Authors:** Abbas Agaimy, Josephine K. Dermawan, Florian Haller, Sabine Semrau, Norbert Meidenbauer, Robert Stoehr, Sigurd Lax, Arndt Hartmann, Ying S. Zou, Deyin Xing, Lars Tögel, John M. Gross, Michael Michal

**Affiliations:** 1 Institute of Pathology, Erlangen University Hospital, Friedrich Alexander University of Erlangen-Nuremberg, Erlangen, Germany; 2 Comprehensive Cancer Center, European Metropolitan Area Erlangen-Nuremberg (CCC ER-EMN), Erlangen, Germany; 3 Department of Pathology and Laboratory Medicine, Cleveland Clinic, Cleveland, Ohio USA; 4 Department of Radiation Oncology, University Hospital, Friedrich-Alexander-University Erlangen-Nürnberg (FAU), Erlangen, Germany; 5 Department of Internal Medicine 5-Hematology and Oncology, Erlangen University Hospital, Friedrich Alexander University of Erlangen-Nuremberg, Erlangen, Germany; 6 Department of Pathology, Hospital Graz II, Academic Teaching Hospital of the Medical University Graz, Graz, Austria; 7 School of Medicine, Johannes Kepler University Linz, Linz, Austria; 8 Department of Pathology, The Johns Hopkins University School of Medicine, Baltimore, Maryland USA; 9 Department of Pathology, Faculty of Medicine in Plzeň, Charles University, Plzeň, Czech Republic; 10 Biotical Laboratory, Ltd, Plzeň, Czech Republic

**Keywords:** Undifferentiated uterine sarcoma, Molecular profiling; nerve sheath tumor, EGFR, Tyrosine kinase fusions, Targeted therapy

## Abstract

With the increasing use of innovative next generation sequencing (NGS) platforms in routine diagnostic and research settings, the genetic landscape of uterine sarcomas has been dynamically evolving during the last two decades. Notably, the majority of recently recognized genotypes in uterine sarcomas represent gene fusions, while recurrent oncogene mutations of diagnostic and/ or therapeutic value have been rare. Recently, a distinctive aggressive uterine sarcoma expressing S100 and SOX10, but otherwise lacking diagnostic morphological, immunophenotypic and molecular features of other uterine malignancies has been presented in a scientific abstract form (USCAP, 2023), but detailed description and delineation of the entity is still missing. We herein describe two high-grade unclassified uterine sarcomas characterized by spindle to round cell morphology and diffuse expression of S100 and SOX10, originating in the uterine body and cervix of 53- and 45-year-old women and carrying an *ERBB3* (p.Glu928Gly) and an *ERBB2* (p.Val777Leu) mutation, respectively. Both tumors harbored in addition genomic *HER2* amplification, *ATRX* mutation and *CDKN2A* deletion. Methylation studies revealed a methylome most similar to MPNST-like tumors, but distinct from melanoma, MPNST, clear cell sarcoma, and endometrial stromal sarcoma. Case 1 died of progressive peritoneal metastases after multiple trials of chemotherapy 47 months after diagnosis. Case 2 is a recent case who presented with a cervical mass, which was biopsied. This study defines a novel heretofore unrecognized aggressive uterine sarcoma with unique phenotypic and genotypic features. Given the potential value of targeting HER2, recognizing this tumor type is mandatory for appropriate therapeutic strategies and for better future delineation of the entity.

## Introduction

During the last two decades, significant progress has been made in elucidating the genetic landscape of uterine mesenchymal neoplasms, with delineation of novel entities and characterization and expansion of the molecular drivers in the spectrum of established neoplasms. Notably, most of these recent advances concern identification of fusion genes [1]. For example, *PLAG1* and *PGR* fusions have been recognized as genetic drivers in subsets of high-grade uterine sarcomas characterized by prominent myxoid and epithelioid/rhabdoid features, respectively, some of which were previously classified as leiomyosarcomas [2, 3]. On the other hands, *ALK* fusions have been confirmed in inflammatory myofibroblastic tumors [4]. In the spectrum of fibrosarcoma-like malignancies, a variety of recurrent tyrosine kinase fusions have allowed identification of distinct molecularly defined entities, including *NTRK* [5], *COL1A1::PDGFB* [5], and others. Uterine tumors resembling ovarian sex cord tumors (UTROSCT) represent another tumor with recently characterized recurrent fusion genes involving *ESR1, GREB1*, *NCOA1-3*, and others [6].

The molecular landscape of endometrial stromal neoplasms has emerged in both the low-grade (LG-) and high-grade (HG-) endometrial stromal sarcoma (ESS) categories [1]. Moreover, a new sarcoma category overlapping with ESS and harboring recurrent *KAT6A/B::KANSL1* fusions has been recently identified [7]. Correctly identifying and precisely subtyping these emerging entities is mandatory for proper risk stratification, which is the basis for optimized treatment and follow-up strategies [8]. We herein describe the detailed clinicopathological and molecular findings of a novel uterine sarcoma characterized by a neurogenic-like (S100/SOX10-positive) immunophenotype and activating mutations involving members of the epidermal growth factor receptor (EGFR/ERBB) family of tyrosine kinases *ERBB3* (*HER3*) and *ERBB2* (*HER2*) to alert pathologists to this rare but possible underdiagnosed entity with therapeutic implications.

## Materials and methods

The cases were identified in our consultation files. The tissue specimens were fixed in formalin and processed routinely for histopathology. Due to the consultation nature of the cases, immunohistochemistry (IHC) was performed in different laboratories and the stains applied varied from case to case, based on tissue availability and initial differential diagnostic considerations (details of the staining protocols and antibody sources are available upon request).

### Targeted RNA Next generation sequencing

For Case 1, RNA was isolated from formalin-fixed paraffin embedded (FFPE) tissue sections and subjected to targeted sequencing using the TruSight RNA Fusion panel (Illumina, Inc., San Diego, CA, USA) as described previously [7]. To analyze the mutational status of commonly cancer related genes, DNA was isolated from FFPE tissue sections using the Maxwell 16LEV Blood DNA kit (Promega, Madison, USA) and submitted to hybrid-capture enrichment-based sequencing analysis using the QIAseq Targeted Human Comprehensive Cancer Panel according to manufacturer’s instructions. Bioinformatic evaluation of the sequencing data, including variant calling and annotation, was done with the CLC Genomics Workbench (QIAGEN, Redwood City, CA, USA). Low quality variants with a score under 200 were filtered out, as well as variants in non-protein-coding regions, synonymous variants, and those present in gnomAD with an allele frequency of over 1%. The variants were assessed for pathogenicity according to ACMG/AMP criteria [9]. Fluorescence in-situ hybridization (FISH) was performed using split apart probes designed to detect translocation involving the *YWHAE, EWSR1* and *FUS* gene loci according to the manufacturer´s instructions. *HER2* copy number status was validated using a color FISH probe (all probes from ZytoVision, Bremerhaven, Germany).

### Methylation studies

For methylation studies, genomic DNA was extracted from formalin-fixed paraffin-embedded tissue sections for each of the samples. Next, 250 ng of genomic DNA was subjected to bisulfite conversion and processed on the Illumina Infinium Methylation EPICv2 platform with over 930,000 methylation sites according to the manufacturer’s instructions [10]. The raw idat files were uploaded to a DNA methylation-based classification tool Sarcoma classifier v10.1 available via https://www.molecularneuropathology.org/ [11, 12]. Additionally, IDAT processing and data analysis on both samples was performed using R version 4.4.0 (RRID:SCR_001905) and the “minfi” package (RRID:SCR_012830). Normalization was performed using the “preprocessSWAN” function and probes with a detection P value > 0.01 were filtered, as were SNP-related probes, and probes on sex chromosomes. Methylation levels were measured using beta values. CpG probes were annotated to the human reference genome using the “IlluminaHumanMethylationEPICv2anno.20a1.hg38” R packages. Unsupervised hierarchical clustering of the top 2,000 most variable CpGs and heatmap generation were performed using the “pHeatmap” package (RRID:SCR_016418) with Euclidean distance for clustering of rows and columns and Ward.D2 for clustering method. Methylation profiles of the *ERBB2/3-*mutated tumors were compared to those of melanoma (12 cases), MPNST (19 cases), MPNST-like tumors (7 cases), clear cell sarcoma (CCS) (7 cases), low-grade (16 cases) and high-grade (9 cases) endometrial stromal sarcomas (LGESS, HGESS) retrieved from NBCI Gene Omnibus (accession # GSE140686) [12].

## Results

### Clinical history of Case 1

A 53-year-old female underwent uterine curettage with a diagnosis of high-grade endometrial stromal, sarcoma (HGESS). She received total abdominal hysterectomy and bilateral salpingo-oophorectomy, followed by adjuvant radiotherapy. The initial diagnosis was revised to probable embryonal rhabdomyosarcoma and then again to HGESS at different institutions. Eleven months later, she presented with multiple peritoneal metastases, for which she received multiple surgeries. Due to new liver metastases developing 16 months later, she was treated with Adriamycin (3 × 25 mg/m^2)^ and Ifosfamide (3 × 2500 mg/m^2^), but the dose was then reduced in the following cycles due to side effects. She then received 9 cycles of trabectedin monotherapy (1.5 mg/m^2^ every 4 weeks), which resulted in short-term improvement, followed soon by disease progression. Thereafter, she received 6 cycles of gemcitabine monotherapy with interruption due to drug intolerance. The patient finally died of progressive systemic metastases (peritoneum, lung, liver, spleen) 47 months from initial diagnosis.

### Pathological findings in Case 1

**Fig. 1:**
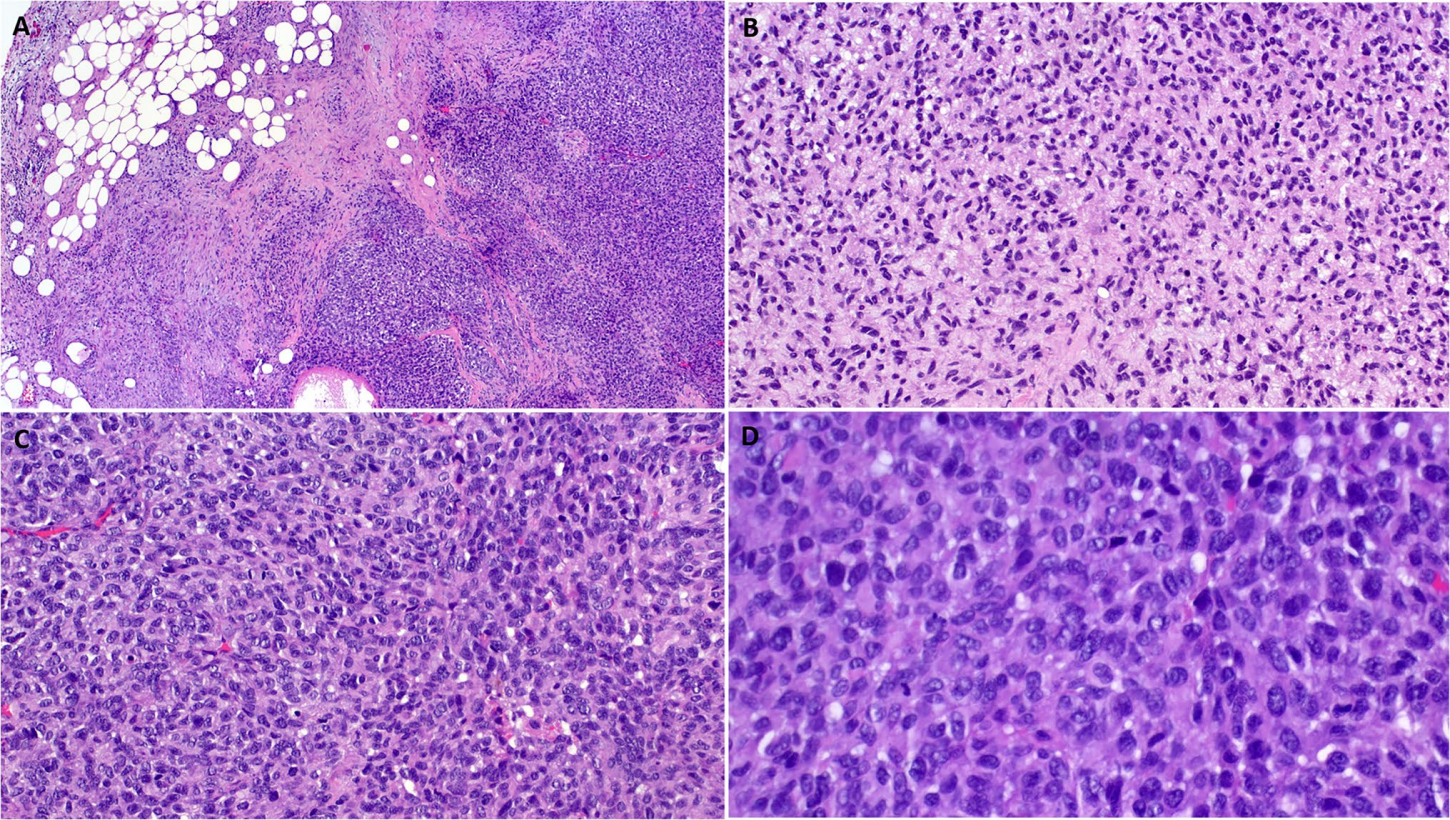
Representative examples of the histological findings in Case 1. **A**: peritoneal metastasis showing solid neoplasm infiltrating the fat tissue. **B**: less cellular spindle cell areas with slightly myxoid appearing stromal background. **C**: diffuse sheets of ovoid to fusiform cells lacking any specific features of ESS. **D**: higher magnification shows small round to ovoid cells with scanty cytoplasm and brisk mitotic activity

**Fig. 2:**
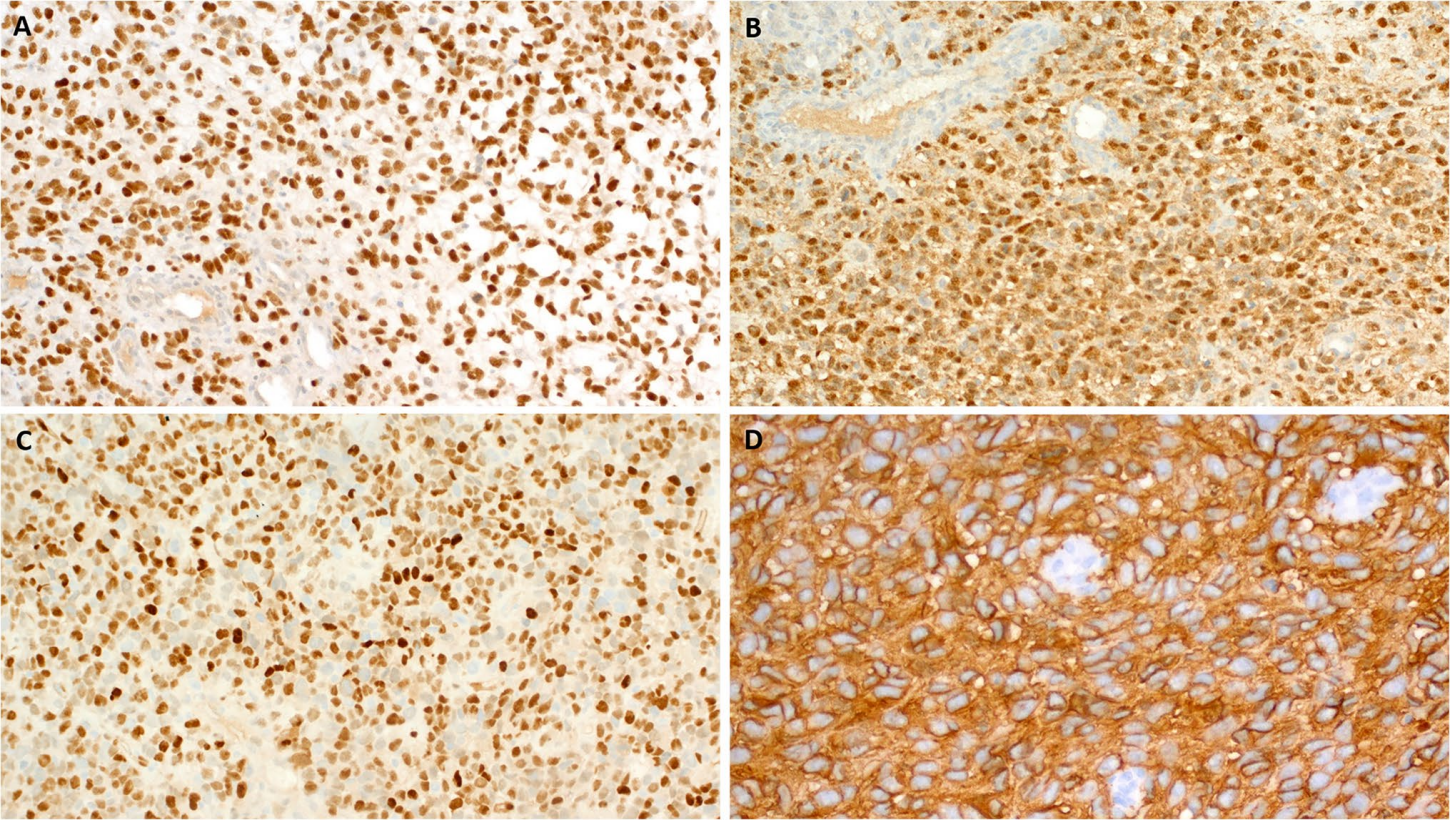
Representative examples of the immunohistochemical findings in Case 1. **A**: diffuse strong nuclear expression of SOX10. **B**: diffuse nucleocytoplasmic reactivity with S100. **C**: moderate diffuse nuclear expression of Cyclin D1. **D**: strong circular membranous expression of HER2

Histological examination of the primary tumor and the multiple resected metastases revealed a high-grade infiltrating neoplasm composed of medium-sized oval to slightly elongated neoplastic cells disposed into diffuse non-cohesive solid sheets within sparse fibrous stroma (Fig. 1A, B, C). The nuclei contained heterogeneous chromatin with variably recognizable nucleoli surrounded by a moderate rim of pale-eosinophilic cytoplasm. Mitotic activity was brisk with 36 mitoses in 10 HPFs (Fig. 1D). Foci of necrosis were seen focally. There were no spiral-like arterioles or any cytological, architectural, or stromal, features of other well established uterine malignancies. Immunohistochemistry displayed strong reactivity with SOX10 (Fig. 2A), S100 (Fig. 2B) and moderate diffuse expression of cyclin D1 (Fig. 2C). Immunohistochemistry for HER2 revealed strong circular membranous staining in all cells (DAKO score 3 + ; Fig. 2D). All other markers tested were negative (pan-melanoma, HMB45, Melan A, keratin AE1/AE3, desmin, alpha smooth muscle actin, h-caldesmon, CD34, p16, CD117, DOG1, myogenin, CD10, estrogen receptor (ER), progesterone receptor (PR), PAX8, WT1, synaptophysin, chromogranin A, CD99 and PRAME)).

### Molecular results in Case 1

The DNA-based sequencing revealed a gain-of-function point mutation in *ERBB3* (*HER3*)(c.2783A > G; p.Glu928Gly; variant allele frequency: 26%) (Table 1). No *ERBB2* mutation was detected. The *HER2* FISH testing revealed low-level amplification (*HER2*: CEP17 ratio = 2.49). In addition, genomic *HER2* amplification was detected and the same *ATRX* c.792 T > G; p.Tyr264* loss-of-function nonsense mutation was detected in two analyzed samples from abdominal wall and liver metastases with an allele frequency of 20% and 43%, respectively. Deletion of *CDKN2A* was detected. RNA sequencing revealed no detectable gene fusions, and the FISH tests were negative for translocations involving *YWHAE, EWSR1* and *FUS* gene loci (not shown).

**Table 1:** Molecular findings in *ERBB2/3* mutated uterine sarcomas

Genetic findings	Case 1	Case 2
Primary genotype	*ERBB3*: c.2783A > G; p.Glu928Gly	*ERBB2*: c.2329G > T; p.Val777Leu
*ATRX*	c.[792 T > G] p.[Tyr264*]	c.[4957-1G > C] splicing
*NRAS*	Wildtype	p.Gln61Lys
*CDKN2A* (chr9)	Copy number loss	Copy number loss
*CDKN2B* (chr9)	No CNV	Copy number loss
Genomic *HER2*	Copy number amplification	Copy number amplification
*HER2* FISH	Amplified (ratio: 2.49)	NA

*CNV *copy number variation, *NA* not available

### Clinical history of Case 2

A 45-year-old female presented with irregular uterine bleeding and was found clinically to have a 0.9 cm cervical polyp that was biopsied and sent out for expert pathologic consultation. She had otherwise no pertinent clinical history or history of another malignancy. This is a recent case and complete surgical excision and referral to a multidisciplinary sarcoma team was recommended.

### Pathological findings in Case 2

**Fig. 3:**
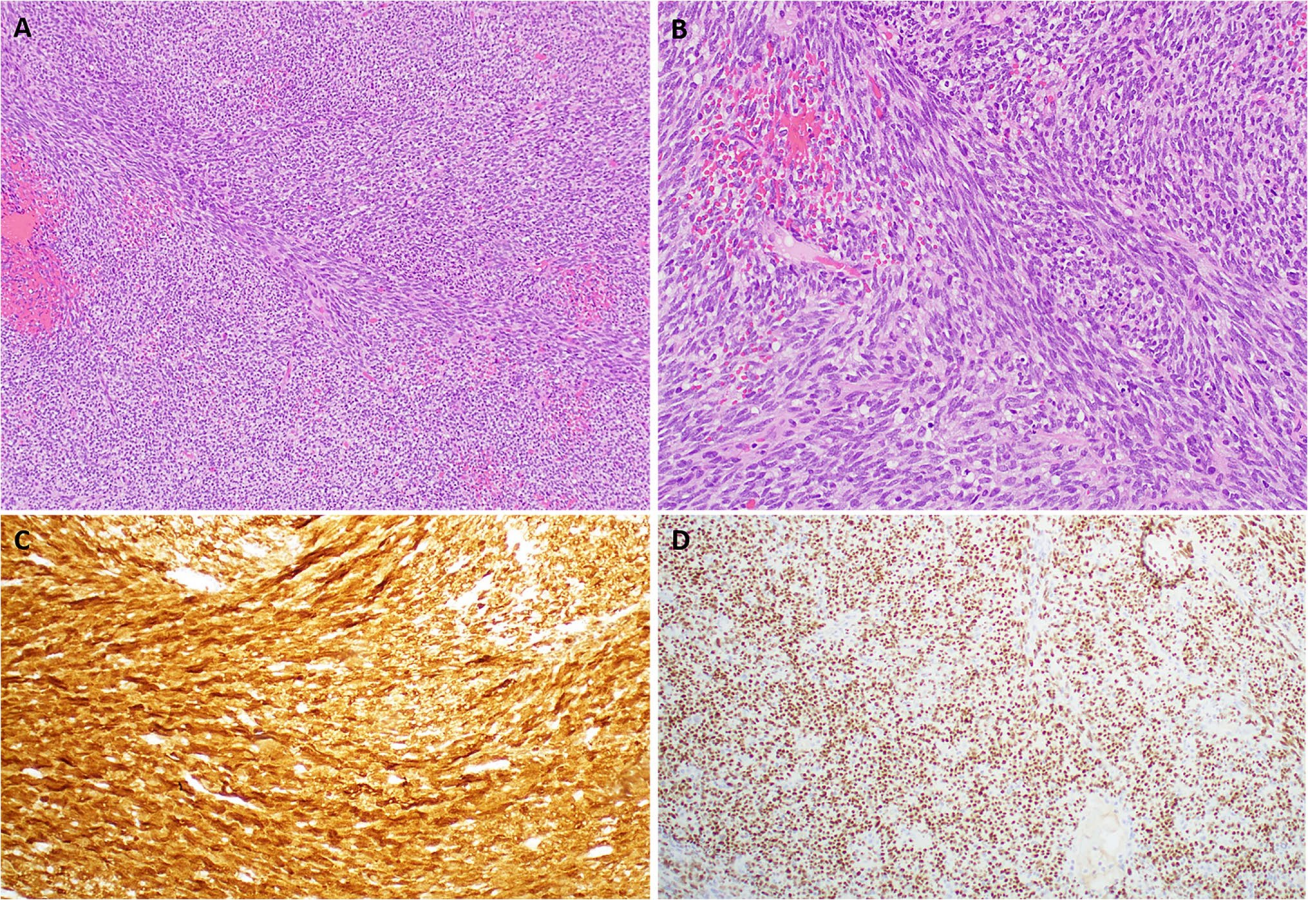
Representative examples of the histological and immunohistochemical findings in Case 2. **A**: diffuse solid sheets and fascicles of monotonous spindle cells imparting a “fibrosarcoma-like” pattern. **B**: higher magnification of **A**. **C**: diffuse nucleocytoplasmic reactivity with S100. **D**: strong nuclear expression of SOX10

Histological examination of the biopsy tissue revealed a highly cellular spindle cell neoplasm composed of monomorphic ovoid to spindle cells with hyperchromatic nuclei and sparse pale-eosinophilic indistinct cytoplasm disposed into compact fascicles and diffuse solid sheets recapitulating the pattern of so-called adult-type fibrosarcoma (Fig. 3A). The cytology of the neoplastic cells was uniform with little nuclear pleomorphism (Fig. 3B). Admixed inflammation is completely absent. Mitotic activity was high with 9 mitoses in 10 HPFs. Immunohistochemistry revealed strong and diffuse expression of S100 (Fig. 2C), SOX10 (Fig. 2D), CyclinD1 and cytoplasmic CD99. CD10 showed focal/patchy expression. Negative markers included ER, CK AE1/AE3, EMA, Melan-A, HMB45, MITF, desmin, Myo D1, smooth muscle myosin, CD117, p40 and CD34.

### Molecular results in Case 2

DNA-based testing revealed multiple pathogenic mutations including an *ERBB2* (*HER2*; c.2329G > T; p.Val777Leu; variant allele frequency: 39%) mutation, a *NRAS* Gln61Lys mutation, and an *ATRX* splicing mutation (c.4957-1G > C) (Table 1). Moreover, deletions of *CDKN2A* and *CDKN2B* were detected. RNA sequencing and FISH were not performed.

### Methylation profiling of Case 1 and 2

**Fig. 4:**
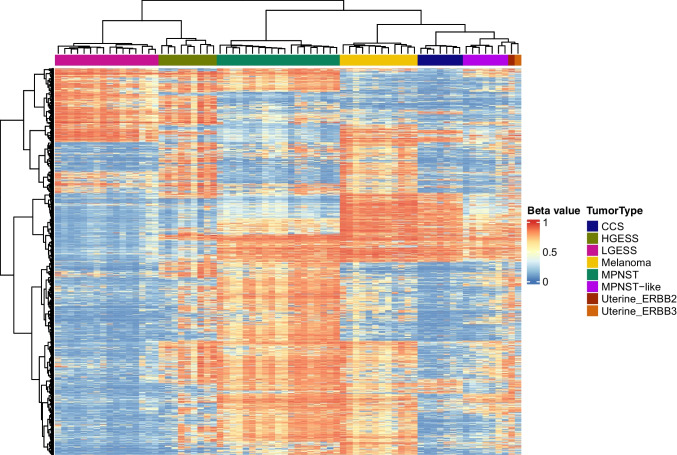
Unsupervised hierarchical clustering of methylation profiles comparing the 2 cases of *ERBB2/3*-mutated sarcomas to melanoma, MPNST, MPNST-like tumors, clear cell sarcoma, low-grade and high-grade endometrial stromal sarcomas

DNA methylation was performed on both cases. The DKFZ soft tissue tumor classifier returned no match for both cases. On unsupervised hierarchical clustering, both cases displayed a methylome that clustered most closely with MPNST-like tumors (Fig. 4).

## Discussion

With the wider application of next generation sequencing modalities in routine practice, the classification of uterine sarcomas has been dynamically evolving [1]. A variety of new entities have been delineated based on precise genotyping and most aggressive malignancies in the historical spectrum of undifferentiated uterine sarcomas could indeed be reclassified by molecular profiling, mostly into the spectrum of HGESS [13]. Most of these recent developments were represented by diverse recurrent oncogene fusions as driver events in distinct subsets of neoplasms of well established (endometrial stromal [1]) origin or of ambiguous (*PLAG1, NTRK* fusions and others [2, 3, 5]) histogenetic origin.

A series of 7 unclassified uterine neoplasms expressing S100 and SOX10, lacking gene fusions and harboring an activating *ERBB2* (*HER2*) mutation at codon 777 were presented in an abstract form at the USCAP annual meeting (Los Angeles, 2023) [14]. Original diagnoses of the presented cases were mostly unclassified or undifferentiated sarcoma with discussion of metastases, MPNST or endometrial stroma sarcoma. Patient’s age range was 42 to 60 years (median, 53). Sites of primary involvement were cervix (*n* = 2), cervix/uterus/vagina (*n* = 2), cervix and uterus (*n* = 1), cervix and pelvis (*n* = 1) and uterus unspecified (*n* = 1). Hysterectomy was the treatment in 3 patients, three received biopsy only and one had removal of the mass with bilateral salpingo-oophorectomy. The tumor stage was stated as T1b (*n* = 1), T2 (*n* = 2), T3b (*n* = 1) and T4 (*n* = 1) [14]. All tumors were cellular with predominance of spindle cells and variable focal or multifocal epithelioid/ round cells with frequent necrosis (3 of 7), focal myxoid stromal changes (3 of 7) and brisk mitotic activity in the range of 1 – 30 mitoses/ 10 HPFs (median, 7). The genomic profiling of the 7 tumors revealed frequent truncating *ATRX* mutations (5 of 7) and homozygous *CDKN2A* (5 of 7) and *CDKN2B* (4 of 7) deletions. *TP53* missense mutations (2 of 7) and *HER2* co-amplification (3 of 7) were observed in subset of tumors [14].

The herein presented cases fit perfectly into this novel tumor category, both histologically, immunohistochemically and molecularly. We detected same *ERBB2* point mutation (p.V777L) in Case 2 as described by Lin et al. [14] and a novel *ERBB3* (*HER3; EGFR3*) point mutation (p.Glu928Gly) in Case 1. Remarkably, a *ATRX* mutation and deletion of *CDKN2A* (p16) together with *HER2* co-amplification at a genomic level were detected in both tumors.

Being a member of the human EGFR family of tyrosine kinases, ERBB2/HER2-neu is an orphan receptor without a known activating ligand, however, it adopts a structure similar to the ligand-bound state and is active in a heterodimeric complex [15]. *HER2* amplifications drive oncogenesis in a significant proportion of aggressive epithelial cancers (carcinomas) including in particular subsets of breast cancer [16] and salivary gland cancer [17] where it is detectable in 15 – 30% of cases.

Valine 777 of HER2 is located in the N-lobe (N-terminal lobe) of the tyrosine kinase domain and results in an elevated kinase activity, an aberrant and enhanced phosphorylation of downstream signaling molecules in cell culture experiments, as well as an increased and more rapid tumor growth compared to wild-type HER2 in xenograft models [18]. Mutation of Valine at position 777 represent one of the frequently detected *HER2* alterations in cancer and is observed in approximately 4.0% of all *HER2* mutated cases [19].

ERBB3 (Erb-B2 Receptor Tyrosine Kinase 3, synonym: HER3) belongs to the EGFR family of receptor tyrosine kinases. Despite the identification of neuregulin-1 (NRG1) as specific ligand, ERBB3 is lacking a significant intrinsic kinase activity and has been considered as kinase dead receptor. However, this paradigm has been continuously challenged and, in fact, the role of ERBB3 in pathogenesis of different malignancies has been demonstrated (as summarized in Black et al. [20]).

The c.2783A > G *ERBB3* mutation replaces glutamic acid, a polar, negatively charged amino acid, by glycine with nonpolar and neutral properties at position 928 in the kinase domain. Glutamic acid at position 928 is implicated in the dimer formation interface of ERBB3 and, in fact, ERBB3 displays oncogenic potential when co-expressed with ERBB2 [21]. In this HER3/HER2 heterodimer, E928G increases the catalytic activity of HER2, an effect which was less pronounced when glutamine acid at position 928 was replaced with Alanine or Lysine, implicating varying functional effects of particular residues at this position [22]. Of note, in a heterodimeric complex, instead of reactivating catalytic activity of ERBB3, the E928G mutation rather increases the dimerization affinity of ERBB3 thereby enhancing its allosteric activation potential [23]. This highlights the necessity of including a functional, catalytical active dimerization partner in the heterodimer, for *ERBB3* E928G to execute its full oncogenic potential. Our Case 1 falls into this pathogenetic category and represents a novel observation in this type of uterine sarcomas, indicating that mutant *ERBB3* concurrent with *ERBB2* amplification represents a novel mechanism driving oncogenesis in tumors lacking the V777L *ERBB2* mutation.

The exact nosology of this neoplasm remains enigmatic. The lack of any detectable gene fusion and the strong and homogeneous expression of SOX10 and S100 exclude all known genetic subtypes of high-grade endometrial stromal sarcoma (HEESS) [1]. In this regard, the diffuse Cyclin D1 expression noted in both of our cases represents a diagnostic pitfall, given that this marker has been proposed as a surrogate for HGESS harboring *YWHAE* and *BCOR* gene fusions [24]. On the other hand, the expression of SOX10 and S100 makes the possibility of an MPNST and a metastatic melanoma important considerations. MPNST-like tumors have been reported in the female genital tract [25]. Their neurogenic nature was favored on the basis of patchy expression of S100 in addition to CD34 reactivity [25]. However, most of these tumors have been recently reclassified as genetically defined tyrosine kinase fusion associated sarcomas including in particular *NTRK* and *COL1A1::PDGFB* fusions [5]. Variable reactivity for S100 is a common feature in *NTRK* fusion sarcomas/neoplasms. However, these fusion tumors lack SOX10 expression, in line with a non-neurogenic and non-melanocytic origin. Indeed, coexpression of S100 (usually variable and patchy) and CD34 represents a valuable clue to tyrosine kinase fusion associated mesenchymal neoplasms originating at different anatomic sites [10]. In the context of non-epithelial and non-myoepithelial neoplasia, coexpression of S100/SOX10 is considered specific for a schwannian or melanocytic line of differentiation. In this regard, the detection of a *NRAS* mutation at position 61 in one of our cases represents a pitfall as this genotype is noted in 20—40% of melanomas, the frequency varying with the clinicopathological tumor type [26]. In our cases, the tumor did not cluster with MPNST or melanoma arguing against both possibilities. Moreover, the distinctive genotype with activating *HER2* mutation, *CDKN2A* deletion and truncating *ATRX* mutation, which are identical to those reported in the cited abstract [14], all argue for a distinctive entity unrelated to melanoma, MPNST, or any of the established sarcoma types of the female genital tract or the soft tissues.

The literature on *HER2* mutations in mesenchymal neoplasms is limited. Ronellenfitsch et al. reported activating *ERBB2* mutations (p.Leu755Ser, p.Asp769Tyr, p.Val777Leu) in 3 of 7 (43%) patients with schwannomatosis, but in none of 8 NF2-asscoiated or sporadic hybrid schwannoma-neurofibroma cases [27]. One schwannomatosis patient had three tumors all harboring the same V777L (p.Val777Leu) *ERBB2* mutation as reported in our cases. We have recently observed a similar mutation (p.Val777Leu) in a case of multiple hybrid schwannoma-neurofibromas unassociated with clinically recognizable NF1 or NF2 syndrome (Agaimy, unpublished data). These reported *ERBB2* kinase domain mutations are known to occur in breast cancer and rarely in carcinomas of other organs and are treatable by pan-ERBB2 inhibitors [28–31].

Finally, Lim et al. have recently reported a high-grade uterine sarcoma carrying a novel *ERBB4* fusion (fused to *CIQTNF1*) in a 49-year-old woman [32]. The tumor morphology was suggestive of HGESS with variable expression of desmin, ER, PR, AE1/3 and cyclin D1 [32]. This report and our current cases point to an emerging role of members of the human EGFR family of tyrosine kinases (fusions and mutations) in the oncogenesis of rare uterine sarcomas.

In summary, we herein describe the first detailed study of a novel *ERBB2/ ERBB3*-mutated S100/SOX10-positive unclassified highly aggressive uterine sarcoma type. The histogenesis of this tumor, its appropriate classification and the potential benefit of targeting the underlying *ERBB2/ ERBB3* tyrosine kinase mutation remain to be verified in the future. Inclusion of SOX10 in high-grade unclassified gynecological sarcomas would be a valuable and cheap screening tool to enhance recognition of this entity, particularly in putative cases of undifferentiated uterine sarcomas.

## Data Availability

The datasets generated during and/or analyzed during the current study are not publicly available, but are available from the corresponding author on reasonable request.

## Declarations

Samples were used in accordance with ethical guidelines for the use of retrospective tissue samples provided by the local ethics committee of the Friedrich-Alexander University Erlangen-Nuremberg (ethics committee statements 24.01.2005 and 18.01.2012).
